# The Nitrilimine–Alkene Cycloaddition Regioselectivity Rationalized by Density Functional Theory Reactivity Indices

**DOI:** 10.3390/molecules22020202

**Published:** 2017-01-26

**Authors:** Giorgio Molteni, Alessandro Ponti

**Affiliations:** 1 Dipartimento di Chimica, Università degli Studi di Milano, via C. Golgi 19, 20133 Milano, Italy; giorgio.molteni@unimi.it; 2 Istituto di Scienze e Tecnologie Molecolari, Consiglio Nazionale delle Ricerche, via C. Golgi 19, 20133 Milano, Italy

**Keywords:** 1,3-dipolar cycloaddition, nitrilimine, alkene, regioselectivity, conceptual density functional theory, reactivity indices, softness

## Abstract

Conventional frontier molecular orbital theory is not able to satisfactorily explain the regioselectivity outcome of the nitrilimine–alkene cycloaddition. We considered that conceptual density functional theory (DFT) could be an effective theoretical framework to rationalize the regioselectivity of the title reaction. Several nitrilimine–alkene cycloadditions were analyzed, for which we could find regioselectivity data in the literature. We computed DFT reactivity indices at the B3LYP/6-311G(2d,p)//B3LYP/6-31G(d,p) and employed the grand potential stabilization criterion to calculate the preferred regioisomer. Experimental and calculated regioselectivity agree in the vast majority of cases. It was concluded that predominance of a single regioisomer can be obtained by maximizing (i) the chemical potential difference between nitrilimine and alkene and (ii) the local softness difference between the reactive atomic sites within each reactant. Such maximization can be achieved by carefully selecting the substituents on both reactants.

## 1. Introduction

**Figure 1:**
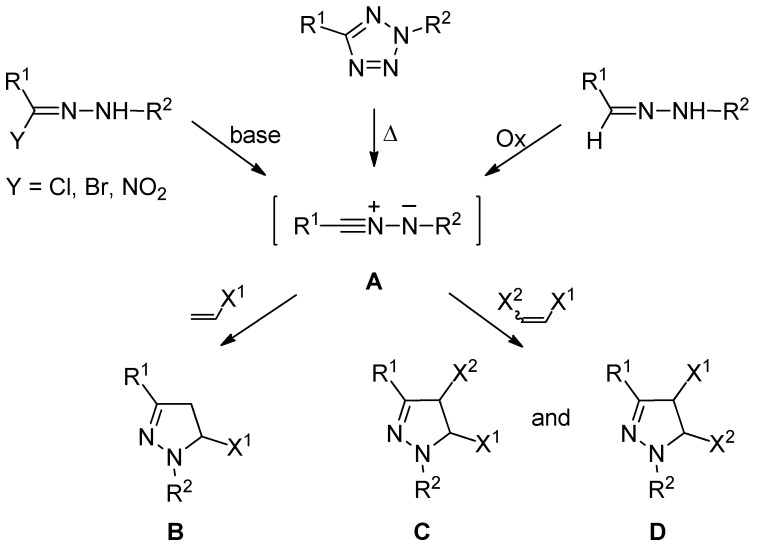
General picture describing the formation of nitrilimine intermediate (**A**) and their regioselectivity towards mono- (**B**) and 1,2-disubstituted (**C**,**D**) alkenes.

Nitrilimines are 1,3-dipolar species which belong to the class of nitrilium betaines [1]. Except for a few examples [2,3], these are labile intermediates [4] which can be generated in situ by the three ways depicted in Figure 1, namely: (i) base-promoted dehydrohalogenation of hydrazonoyl halides or nitrohydrazones; (ii) thermolysis of 2,5-disubstituted tetrazoles; and (iii) oxidation of aldehyde hydrazones [5]. Cycloaddition of the reactive nitrilimine intermediate **A** towards an array of unsaturated species gives rise to a variety of five-membered heterocycles [5]. Focusing to the alkenes as the dipolarophilic counterpart, their cycloaddition with **A** represents a general method for the synthesis of 4,5-dihydropyrazoles [6]. It can be easily argued that the relative orientation of the reacting species implies a regioselectivity problem. As far as monosubstituted alkenes are concerned, the nitrilimine cycloaddition with both electron-rich and electron-poor dipolarophiles generally provides 5-substituted-4,5-dihydropyrazoles (in the following: 5-substituted pyrazolines) **B** as the unique regioisomer. This is the case of aryl-, alkyl-, alkoxy-, alkoxycarbonyl-, and amino-alkenes [5].

The current view to explain the nitrilimine–alkene cycloaddition regioselectivity is based upon the perturbation approach (frontier molecular orbital (FMO) theory) [7,8]. Within this frame, cycloadditions to electron-rich alkenes are LUMO-dipole controlled giving rise to 5-substituted pyrazolines [7]. On the other hand, in the case of electron-poor alkenes, a dominant HOMO-dipole control should be at work giving a prevalence of 4-substituted pyrazolines. Since such a prevision is in contrast with experimental facts, an explanation accounting for the formation of 5-substituted pyrazolines has been proposed assuming that steric requirements should overcome electronic effects [5].

In the case of 1,2-disubstituted alkenes as the dipolarophilic species, nitrilimine cycloaddition generally provides a mixture of the possible pyrazolines **C** and **D** [5,6]. Here again, the FMO theory provides a qualitative rationale based on the assumption that the atomic coefficients of the C=C double bond must have similar values [7].

In light of the above statements, it may appear that the FMO treatment of the nitrilimine–alkene cycloaddition regioselectivity looks somewhat puzzling since ad hoc assumptions are required to rationalize the experimental results. Thus, a theoretical frame able to predict the regioselectivity of the nitrilimine–alkene cycloaddition should be needed since, to the best of our knowledge, no improvement over the FMO treatment of this subject has been reported yet. A direct approach to the problem would encompass locating the regioisomeric transition states (TSs) and comparing the difference in electronic or Gibbs energy with the experimental regioselection. These energies should be computed at the highest affordable level of theory and basis set, possibly including solvent effects. Such a direct approach would provide accurate results, which however would be based on the global molecular energies. For the synthetic chemist, the results would be difficult to transfer to other reactions and would not provide further or novel insight in the structure/regioselectivity relationship.

We therefore considered using the tools provided by conceptual density functional theory (DFT) [9,10], a theoretical framework which made it possible to rigorously define many chemical quantities (e.g., electronegativity) and to compute them from the molecular energy and electron density. Conceptual DFT provides many reactivity indices summarizing the changes occurring when a molecule accepts or donates electrons during a reaction. Often, both global (molecular) and local reactivity indices can be defined and calculated. For instance, the local softness can be defined as the derivative of the electron density with respect to the electron chemical potential at fixed molecular geometry [11]. Local indices (usually condensed to atomic indices) form a practical toolkit to study the reactivity at different sites within a molecule and provide results expressed in a language appealing to the synthetic chemist.

Regioselectivity is clearly amenable to investigation based on local (atomic) reactivity indices and literature offers many examples of regioselectivity of 1,3-dipolar cycloadditions (1,3-DCs) that were successfully explained using DFT-based reactivity indices [12]. A general criterion to point out the preferred regioisomer resulting from an addition reaction has been derived by minimizing the grand potential Ω [13]. This approach, which is based on the energy and electron density of the reactants only and does not require the calculation of the TSs, was applied to the quantitative study of the regioselectivity of the 1,3-DCs of azides [14] and nitrile oxides [15]. This theoretical framework allowed us to rationalize the regioselectivity in the nitrilimine–alkyne [16] and the nitrilimine–allene cycloadditions, too [17]. The present paper is aimed at investigating the regioselectivity for the nitrilimine–alkene cycloaddition within the framework of the conceptual DFT.

## 2. Results and Discussion

**Scheme 1:**
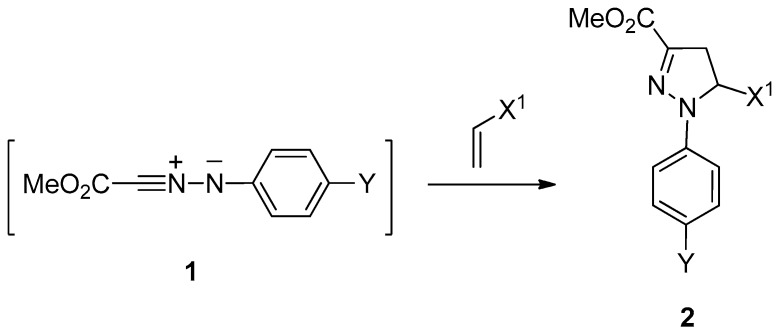
Cycloaddition between nitrilimines **1** and 1-X^1^-ethenes giving 5-(X^1^)-pyrazolines **2**.

A number of regioselectivity data of the nitrilimine–alkene cycloaddition were taken from the seven relevant papers that represent the core of this subject. These data, which are collected in Table 1, Table 2 and Table 3 and accompanied by the corresponding reaction schemes, can be naturally classified into three groups according to the nitrilimine substituents. Reaction group (RG) I comprises reactions between 1-X^1^-ethenes and *C*-methoxycarbonyl-*N*-(4-Y-phenyl)nitrilimines **1** [18]. These reactants yielded the 5-(X^1^)-pyrazolines **2** only (see Scheme 1 and Table 1).

**Scheme 2:**
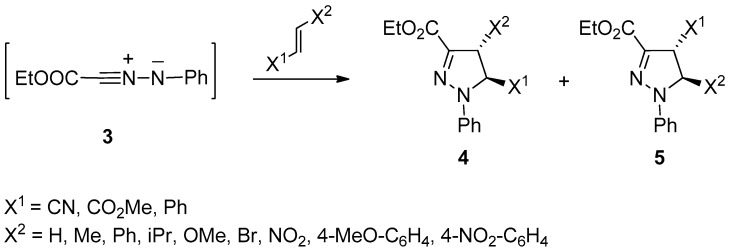
Cycloaddition between nitrilimine **3** and mono- or disubstituted ethenes giving regioisomeric pyrazolines **4** and **5**.

In RG-II we collated data regarding the cycloaddition between *C*-ethoxycarbonyl-*N*-phenyl nitrilimine **3** and several mono- and disubstituted-(*E*)-1,2-ethenes [19] (see Scheme 2 and Table 2). Again, 1,3-DCs in RG-II yielded selectively the 5-(X^1^)-pyrazolines **4** in the case of 1-X^1^-ethenes. Using disubstituted chalcone, (Table 2, entry 9), a mixture of pyrazolines **4** and **5** was obtained. Since we were surprised that the 1,3-DC of **3** with ethyl crotonate (Table 2, entry 7) [19] was reported to give pyrazoline **4** only, we performed the new cycloaddition between ethyl crotonate and **1a**, which gave a regioisomer mixture (Table 2, entry 8). So, the datum previously reported for the cycloaddition between ethyl crotonate and **3** should be considered incorrect (see also the regioselectivity outcome of methyl crotonate with diphenylnitrilimine **6**, Table 3, entry 2).

**Scheme 3:**
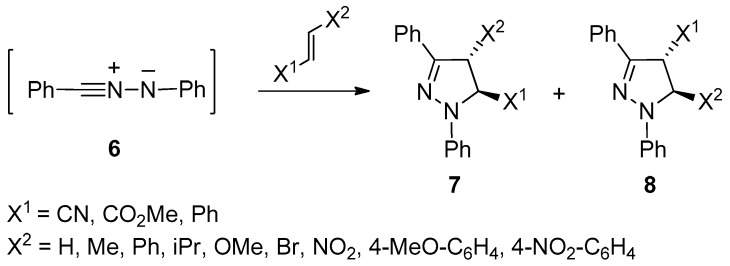
Cycloaddition between diphenylnitrilimine **6** and mono- or disubstituted ethenes giving regioisomeric pyrazolines **7** and **8**.

Finally, RG-III comprises the reactions of diphenylnitrilimine **6** with acrylonitrile (X^1^ = CN) [20,21] and several disubstituted (*E*)-1,2-ethenes [22,23,24] (see Scheme 3 and Table 3). All reactions in this group yielded both regioisomers **7** and **8** spanning a largely different product ratio.

We now turn to the analysis of these regioselectivity data using local DFT-based reactivity indices. Due to the large number of molecular systems investigated, we only present the data strictly needed for the discussion.

In Table 4, Table 5 and Table 6 the chemical potential μ and the global softness *S* of the monosubstituted and (*E*)-1,2-disubstituted ethenes and of nitrilimines **1**, **3** and **6** are reported. The substituent effect on μ is significant and agrees with common chemical knowledge (electron donating/accepting substituents increase/decrease μ). The monosubstituted ethenes and propenes (X^1^ = Me) are less soft than the nitrilimines but the substituted styrenes (X^1^ = Ph) and stilbenes (X^1^ = Ph, X^2^ = aryl) are about as soft as the nitrilimines, evidencing the role of aryl rings in making molecules softer.

When basing on the grand potential criterion to explain regioselectivity [13], the first step is to classify the reacting molecules as electron-donating or -accepting in order to use the local softness for nucleo- or electro-philic attack, respectively. We carried out this classification by considering the electron chemical potential of the reactant molecules since we are interested in the direction of the electron transfer at the very beginning of the 1,3-DC, before that electron reshuffling becomes important [25]. This initial step has been shown to determine the regioselectivity in 1,3-DCs [14,15,16,17]. Of course, we are aware that other global DFT indices have been successfully proposed to describe the molecular ability to donate or accept electrons, such as Parr’s electrophilicity index ω [26], the electroaccepting ω^+^ and electrodonating ω^−^ powers [27], and the nucleophilicity index *N* [28]. However, these indices were proposed and applied as reactivity descriptors rather than indices showing the direction of the initial electron transfer. For instance, Parr’s ω was successfully employed to classify electrophiles in 1,3-DC reactions [29] and shown to be closely related to the computed activation energy of the Diels–Alder reaction of ethenes with cyclopentadiene [30]. The nucleophilicity index *N* was also employed for classification [31] and shown to closely correlate to nucleophilic rate constants [32].

The direction of electron transfer was thus evaluated on the basis of the electron chemical potential, as shown in Table 7, Table 8 and Table 9 where the μ(nitrilimine)—μ(ethene) differences are listed. A positive difference means that the nitrilimine donates electrons to the ethene. The direction of electron transfer depends on the substituents on both alkene and nitrilimine, as expected on the basis of the electron demand of the substituents. It is noteworthy that in each reaction group, cases where the nitrilimine acts as nucleophile or electrophile can be found.

When 1-X^1^-ethenes and nitrilimines **1** are considered (RG-I, Table 7), the direction of the electron transfer is mainly dictated by the nitrilimine Y substituent but the effect of the ethene X^1^ substituent is also significant. In RG-II and -III (Table 8 and Table 9), the effect of the alkene substituents can be more clearly seen since a single nitrilimine is used as a substrate in each RG.

The electrophilic *s*^+^ or nucleophilic *s*^−^ local softness is chosen for each reactant pair consistently with their chemical potential difference and condensed to individual atoms using Hirshfeld population analysis [33]. The grand potential stabilization ΔΩ can now be computed for the pathways leading to the two possible regioisomers. The grand potential stabilization difference δΔΩ = ΔΩ(5-X^1^) − ΔΩ(4-X^1^) between the pathways leading to regioisomers **5** and **4** is reported in Table 10, Table 11 and Table 12. A negative δΔΩ indicates that the 5-(X^1^)-pyrazoline regioisomer is favored (see Section 3. Computational Methods).

All reactions in RG-I yielded the 5-X^1^-substituted-4,5-dihydropyrazole **2** only, though with largely different yield (see Table 1 and Scheme 1). Such experimental outcome is correctly predicted by the computed δΔΩ difference, which is negative for all reactions in RG-I. The agreement should however be considered qualitative since the very large experimental regioselection towards **2** corresponds to both reasonably large values (reactions where X^1^ and Y have opposite electron demand) and very low values of δΔΩ (reactions where X^1^ and Y have similar electron demand). Such large variation in the computed δΔΩ differences can be traced back to the corresponding large variation in chemical potential difference between reactants. For instance, μ in butylvinylether (X^1^ = *n*-OBu) and *C*-methoxycarbonyl-*N*-(4-methoxyphenyl)nitrilimine **1b** differ by as little as 4 meV (see Table 7) and make δΔΩ very small. Recall also that the chemical potential difference appears squared in the expression for δΔΩ (see Equation (6)). These results seem less satisfying than our previous investigation of the nitrilimine–alkyne cycloaddition [16], where a quantitative correlation between experimental regioselectivity and δΔΩ was obtained, but it should be noted that RG-I comprises several ethenes with widely different electron demand while we previously considered a single acetylenic dipolarophile, i.e., methyl propiolate. Thus, the present qualitative agreement can be considered satisfactory.

RG-II comprises the 1,3-DCs between nitrilimine **3** and **1a** and several mono- and 1,2-disubstitued ethenes (see Table 2 and Scheme 2). All reactions in this RG were reported to yield the 5-(X^1^)-pyrazoline with the exception of ethyl crotonate (Table 2, entry 8) and chalcone (Table 2, entry 9), which undergo cycloaddition, yielding a product ratio **4**:**5** = 5-X^1^:4-X^1^ = 79:21 and 60:40, respectively. The computed δΔΩ difference is, in any RG-II reaction, negative, in agreement with the experiment. However, as in the RG-I case, we hold this agreement as qualitative since δΔΩ for ethyl crotonate (−0.024 meV) and chalcone (−0.049 meV) are more negative than for 1-hexene (−0.006 meV). Thus, the latter would be expected to display lesser regioselectivity than the former ones but 1-hexene actually yields the 5-X^1^ regioisomer only.

Finally, RG-III comprises the 1,3-DCs of diphenylnitrilimine **6** with acrylonitrile and several 1,2-disubstitued ethenes. This is the most interesting group since extensive quantitative regioselectivity data can be found in the literature. All reactions in RG-III gave a regioisomeric mixture of cycloadducts (see Scheme 3 and Table 3), ranging from **7**:**8** = 5-X^1^:4-X^1^ = 97:3 to 15:85. These data allow us to investigate whether there is a quantitative relationship between δΔΩ and the regioisomeric product ratio. The quantity log_10_(4-X^1^:5-X^1^) is proportional to the difference of the activation energy between the pathways leading to the two regioisomers and is negative when the 5-X^1^ regioisomer is favored, similarly to δΔΩ.

**Figure 2:**
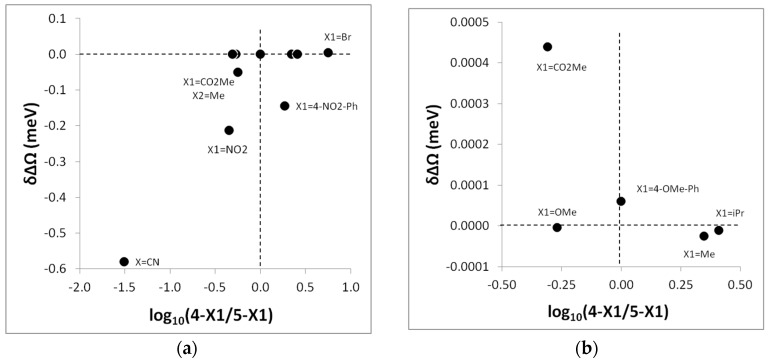
Plot of the grand potential stabilization difference δΔΩ = ΔΩ(5-X) − ΔΩ(4-X) vs. the logarithm of the regioisomeric product ratio log_10_(4-X^1^:5-X^1^) for the 1,3-DC of diphenyl nitrilimine **6** with 1,2-disubstitued ethenes and acrylonitrile (X^1^ = CN). Panel (**b**) is an enlargement of panel (**a**) near the origin of the co-ordinate axes. When not specified, X^2^ = Ph is intended.

Inspection of Table 12 and Figure 2 reveals several details about the 1,3-DC to diphenylnitrilimine **6**. The regioselectivity of the reactions of **6** with electron-poor alkenes acrylonitrile (X^1^ = CN), methyl crotonate (X^1^ = CO_2_Me, X^2^ = Me), bromostyrene (X^1^ = Br, X^2^ = Ph), and nitrostyrene (X^1^ = NO_2_, X^2^ = Ph) are in qualitative agreement with the DFT-based prediction and, moreover, log_10_(4-X^1^:5-X^1^) and δΔΩ are approximately linearly correlated. It should however be noted that the grand potential stabilization criterion fails to predict the regioselectivity of the 1,3-DC between **6** and electron-poor 4-nitrostilbene (X^1^ = 4-NO_2_-Ph, X^2^ = Ph). We currently have no explanation for this failure. In the remaining cases, we note that δΔΩ is very small in contrast with the significant experimental regioselectivity, varying from 5-X^1^:4-X^1^ = 67:33 to = 28:72.

The small δΔΩ values are due to the combination of several effects which are best discussed with reference to Equation (6) (see Section 3. Computational Methods). First, the small softness difference between the two termini of the diphenylnitrilimine [*s*(C_1_) − *s*(N_3_)] and between the *sp*^2^ carbons of substituted styrenes [*s*(C_1_) − *s*(C_2_)] makes δΔΩ small since such softness differences multiply each other in the expression of δΔΩ. These small softness differences, due to the almost symmetric substituents and the electron-reservoir effect of aryl rings, show that the reactivity of the atomic sites within each reactant is very similar. Furthermore, in some cases, the chemical potential difference Δμ between nitrilimine and alkene is also small. This makes δΔΩ even smaller since it is proportional to Δμ^2^. Conversely, when the alkene substitution is single (acrylonitrile) or the strong electron acceptor NO_2_ is present, both the local softness difference between the *sp*^2^ carbons of the alkene and the chemical potential difference increase. Electronic effects thus show up as a sizeable δΔΩ and correlate with log_10_(4-X^1^:5-X^1^).

RG-III nicely shows how substitution affects the electronic factors leading to regioselectivity. Indeed, in the cases where δΔΩ is small, electronic effects related to the initial electron transfer between reactants are weak and scarcely affect regioselectivity. Other electronic effects, such as the “charge reshuffling” term [25] could be responsible for the regioselection. However, considering how crowded pyrazolines **7** and **8** are, it is reasonable to think that steric hindrance effects have a more important role in determining the regioselectivity when the alkene is 1,2-disubstituted.

## 3. Computational Methods

All DFT calculations were performed by means of the GAUSSIAN 09 program suite [34] using the hybrid B3LYP functional. The molecular geometry of the neutral nitrilimines and alkenes was fully optimized using the 6-31G(d,p) basis set by computing the force constants at every optimization step. The geometry of all neutral compounds corresponds to an energy minimum (no imaginary frequencies). The molecular wavefunction of the neutral compounds was computed using the 6-311G(2d,p) basis set at the 6-31G(d,p) geometry. The molecular wavefunction of the mono-cationic and mono-anionic species was calculated using the 6-311G(2d,p) and 6-311G++(2d,p) basis sets, respectively, in both cases at the 6-31G(d,p) geometry of the neutral molecule. Global DFT-based reactivity indices were computed within the finite difference approximation using the energy of the cationic and anionic species to compute the vertical ionization potential (*I*) and electron affinity (*A*), respectively.

(1) $$I=E_{N_{0}-1}-E_{N_{0}};\;A=E_{N_{0}}-E_{N_{0}+1}$$

where *E*(*N*), *N* = *N*_0_ − 1, *N*_0_, *N*_0_ + 1, is the molecular energy of the cationic, neutral, and anionic system, respectively. In this way, the electron density relaxation due to the removal/addition of one electron from/to the molecular system is taken into account. The electron chemical potential μ and the global softness *S* were computed as:

(2) $$\mu =-\frac{I+A}{2};\;S=\frac{1}{I-A}$$

The atomic electron populations *p_i_* were calculated performing Hirshfeld population analysis [33] with hydrogens summed into heavy atoms. The local softness *s* was condensed to individual atoms [35] using Hirshfeld atomic electron populations [36]. The local softness of atom *i* was computed as:

(3) $$s_{I}^{+}=p_{i}(N_{0}+1)-p_{i}\left(N_{0}\right)$$

for a reactant undergoing nucleophilic attack, and as:

(4) $$s_{i}^{-}=p_{i}\left(N_{0}\right)-p_{i}(N_{0}-1)$$

for electrophilic attack, where *p_i_*(*N*), *N* = *N*_0_ − 1, *N*_0_, *N*_0_ + 1, is the electron population of atom *i* in the cationic, neutral, and anionic system, respectively.

Because of the general agreement about the concertedness of 1,3-DCs, we used the maximization of the grand potential stabilization as regioselectivity criterion due to two bond-forming interactions. This principle is a generalization of the hard–soft acid-basis principle and yields a quantitative regioselectivity criterion for 1,3-DCs [13]. Let us denote the grand potential variation ΔΩ for the formation of the 5-X- and 4-X-pyrazoline as ΔΩ(5-X) and ΔΩ(4-X), respectively. Then, the formation of the 5-X-pyrazoline is favored when:

δΔΩ = ΔΩ (5-X) − ΔΩ(4-X) < 0,(5)

which can be rewritten as:

δΔΩ = (1/2) (μ_a_ − μ_n_)^2^*P* {[*s*(a, C_1_) − *s*(a, C_2_)] [*s*(n, C_1_) − *s*(*n*, N_3_)]} < 0 (6)

where μ_a_ and μ_n_ are the chemical potential of the alkene and nitrilimine, respectively, *s*(a, C_1_) is the local atomic softness of carbon C_1_ of the alkene (and similarly for the other local softnesses), and *P* is a non-negative rational function of all local softnesses. Clearly, the sign of Expression (6) only depends on the term in curly braces.

## 4. Conclusions

The analysis of the regioselectivity of the 1,3-DC of mono- and di-substituted alkenes to nitrilimines based on calculated DFT reactivity indices and the grand potential stabilization criterion indicated the preferred regioisomers in agreement with experimental findings in the vast majority of the considered cases. However, the grand potential stabilization criterion is not able to quantitatively predict the regioisomers ratio of nitrilimine–alkene 1,3-DCs, probably because of the ample gamut of analyzed reactions. Of course, our approach is approximate under other respects. For instance, it is assumed that the energy difference between the TSs is only due to the electronic energy while vibrational and solvation effects are neglected. While it is recognized that 1,3-DCs are scarcely affected by solvent effects [37], vibrational (i.e., finite temperature) effects could be another reason for the lack of quantitative agreement between calculation and experiment.

The present analysis showed that achieving a good electronically-controlled regioselectivity sets requirements on both the reactants considered in themselves and the particular reactant pair that will undergo the 1,3-DC. Reactants should have large softness difference between their atoms involved in the bond formation and should be chosen so that they have largely different electron chemical potential. A large intramolecular softness difference between reactive atomic sites is easy to achieve for mono-substituted alkenes; much less so for substituted styrenes and stilbenes and for nitrilimines. A large chemical potential difference between the reactants can be obtained when substituents with opposite electron demand are present on the nitrilimine and alkene.

## Figures and Tables

**Table:** 

	1a	1b	1c	1d	
Y	H	OMe	Me	NO_2_	X^1^ = *n*-BuO, CN, CO_2_Et, *n*-Bu

**Table 1:** Cycloaddition between nitrilimines **1** and 1-X^1^-ethenes (reaction group (RG)-I) giving 5-(X^1^)-pyrazolines **2** (% yields).

X^1^	Y			
	H (1a)	OMe (1b)	Me (1c)	NO_2_ (1d)
*n*-Bu	17	52	31	0
*n*-BuO	15	27	24	10
CO_2_Et	95	93	95	3
CN	56	80	78	0

**Table 2:** Cycloaddition between nitrilimines **3** and **1a** and mono- or disubstituted ethenes giving regioisomeric pyrazolines **4** and **5** (RG-II).

Entry	Nitrilimine	X^1^	X^2^	Yield (%)		Product Ratio
				4	5	4:5
1	**3**	*n*-Bu	H	80	0	100:0
2	**3**	*n*-BuO	H	100	0	100:0
3	**3**	CO_2_Me	H	80	0	100:0
4	**3**	CONH_2_	H	81	0	100:0
5	**3**	CN	H	70	0	100:0
6	**3**	Ph	H	85	0	100:0
7	**3**	CO_2_Et	Me	100	0	100:0
8	**1a**	CO_2_Et	Me	62	16	79:21
9	**3**	COPh	Ph	43	29	60:40

**Table 3:** Cycloaddition between diphenylnitrilimine **6** and mono- or disubstituted ethenes giving regioisomeric pyrazolines **7** and **8** (RG-III).

Entry	X^1^	X^2^	Overall Yield	Product Ratio
			7 + 8 (%)	7:8
1	CN	H	66	97:3
2	CO_2_Me	Me	95	64:36
3	CO_2_Me	Ph	95	67:33
4	Me	Ph	82	28:72
5	*i*-Pr	Ph	98	31:69
6	OMe	Ph	65	65:35
7	Br	Ph	88	15:85
8	NO_2_	Ph	81	69:31
9	4-MeO-C_6_H_4_	Ph	95	50:50
10	4-NO_2_-C_6_H_4_	Ph	74	35:65

**Table 4:** Calculated chemical potential μ and global softness *S* of the investigated 1-X^1^-ethenes.

1-X^1^-Ethenes		
X^1^	μ (eV)	*S* (eV^−1^)
*n*-Bu	−4.41	0.101
*n*-OBu	−4.05	0.112
CO_2_Et	−5.07	0.098
CO_2_Me	−5.10	0.095
CONH_2_	−4.77	0.103
CN	−5.31	0.093
Ph	−3.98	0.118

**Table 5:** Calculated chemical potential μ and global softness *S* of the investigated (*E*)-[1-(X^1^), 2-(X^2^)]-ethenes.

(*E*)-[1-(X^1^), 2-(X^2^)]-Ethenes			
X^1^	X^2^	μ (eV)	*S* (eV^−1^)
Me	CO_2_Me	−4.70	0.099
Me	CO_2_Et	−4.74	0.102
Ph	Me	−3.73	0.121
Ph	*i*-Pr	−3.76	0.124
Ph	OMe	−3.62	0.129
Ph	Br	−4.09	0.125
Ph	COPh	−4.71	0.147
Ph	CO_2_Me	−4.59	0.132
Ph	NO_2_	−5.30	0.137
Ph	4-OMe-Ph	−3.65	0.155
Ph	4-NO_2_-Ph	−4.84	0.170

**Table 6:** Calculated chemical potential μ and global softness *S* of the investigated nitrilimines.

R^1^–C≡N^+^–N^−^-(4-Y-C_6_H_4_)				
Nitrilimine	Y	R^1^	μ (eV)	*S* (eV^−1^)
**1a**	H	CO_2_Me	−4.35	0.143
**1b**	OMe	CO_2_Me	−4.05	0.152
**1c**	Me	CO_2_Me	−4.22	0.146
**1d**	NO_2_	CO_2_Me	−5.24	0.162
**3**	H	CO_2_Et	−4.31	0.143
**6**	H	Ph	−3.82	0.156

**Table 7:** Electron chemical potential difference μ(nitrilimine **1**)–μ(ethene) in eV for the RG-I. Positive values indicate that the nitrilimine transfers electrons to the ethene.

RG-I, MeO_2_C–C≡N^+^–N^−^-(4-Y-C_6_H_4_)				
X^1^	Y			
	H (1a)	OMe (1b)	Me (1c)	NO_2_ (1d)
*n*-Bu	0.06	0.37	0.20	−0.83
*n*-OBu	−0.30	0.004	−0.17	0.07
CO_2_Et	0.72	1.02	0.85	
CN	0.96	1.26	1.09	

**Table 8:** Electron chemical potential difference μ(nitrilimine)–μ(ethene) in eV for the RG-II. Positive values indicate that the nitrilimine transfers electrons to the ethene.

RG-II			
X^1^	X^2^	EtO_2_C–C≡N^+^–N–Ph (3)	MeO_2_C–C≡N^+^–N–Ph (1a)
*n*-Bu	H	0.10	
*n-*OBu	H	−0.26	
CO_2_Me	H	0.79	
CONH_2_	H	0.46	
CN	H	1.00	
Ph	H	−0.33	
CO_2_Et	Me	0.43	0.39
COPh	Ph	0.40	

**Table 9:** Electron chemical potential difference μ(diphenylnitrilimine **6**)–μ(ethene) in eV for the RG-III. Positive values indicate that the nitrilimine transfers electrons to the ethene.

RG-III		
X^1^	X^2^	Ph–C≡N^+^–N–Ph (6)
CN		1.49
CO_2_Me	Me	0.88
Me	Ph	−0.09
*i*-Pr	Ph	−0.06
OMe	Ph	−0.20
Br	Ph	0.27
CO_2_Me	Ph	0.76
NO_2_	Ph	1.47
4-OMe-Ph	Ph	−0.18
4-NO_2_-Ph	Ph	1.02

**Table 10:** Grand potential stabilization difference δΔΩ = ΔΩ(5-X^1^) − ΔΩ(4-X^1^) (meV) for 1,3-DC of nitrilimines **1** to alkenes. Negative values indicate that the 5-X-pyrazoline regioisomer is favored.

RG-I, MeO_2_C–C≡N^+^–N^−^-(4-Y-C_6_H_4_)				
X^1^	Y			
	H (1a)	OMe (1b)	Me (1c)	NO_2_ (1d)
*n*-Bu	−0.002	−0.114	−0.027	−0.341
*n*-OBu	−0.045	−3 × 10^−6^	−0.014	−1.022
CO_2_Et	−0.302	−0.844	−0.479	−0.010
CN	−0.536	−1.211	−0.768	−0.002

**Table 11:** Grand potential stabilization difference δΔΩ = ΔΩ(5-X^1^) − ΔΩ(4-X^1^) (meV) for 1,3-DC of nitrilimines **3** and **1a** to alkenes. Negative values indicate that the 5-X-pyrazoline regioisomer is favored.

RG-II			
X^1^	X^2^	EtO_2_C–C≡N^+^–N–Ph (3)	MeO_2_C–C≡N^+^–N–Ph (1a)
*n*-Bu	H	−0.006	
*n*-OBu	H	−0.036	
CO_2_Me	H	−0.422	
CONH_2_	H	−0.159	
CN	H	−0.577	
Ph	H	−0.061	
CO_2_Et	Me	−0.029	−0.024
COPh	Ph	−0.049	

**Table 12:** Grand potential stabilization difference δΔΩ = ΔΩ(5-X^1^) − ΔΩ(4-X^1^) (meV) for 1,3-DC of diphenylnitrilimine **6** to alkenes. Negative values indicate that the 5-X-pyrazoline regioisomer is favored.

RG-III		
X^1^	X^2^	Ph–C≡N^+^–N–Ph (6)
CN	H	−0.579
CO_2_Me	Me	−0.050
Me	Ph	−2.5 × 10^−5^
*i*-Pr	Ph	−1.1 × 10^−5^
OMe	Ph	−3.9 × 10^−6^
Br	Ph	0.005
CO_2_Me	Ph	4.4 × 10^−4^
NO_2_	Ph	−0.213
4-OMe-Ph	Ph	6.1 × 10^−5^
4-NO_2_-Ph	Ph	−0.144
